# Patient-reported outcome measures in osteoarthritis: a systematic search and review of their use and psychometric properties

**DOI:** 10.1136/rmdopen-2018-000715

**Published:** 2018-12-16

**Authors:** Åsa Lundgren-Nilsson, Anna Dencker, Annie Palstam, Gert Person, Mike C Horton, Reuben Escorpizo, Ayse A Küçükdeveci, Sehim Kutlay, Atilla H Elhan, Gerold Stucki, Alan Tennant, Philip G Conaghan

**Affiliations:** 1 Institute of Neuroscience and Physiology, Sahlgrenska Academy, University of Gothenburg, Gothenburg, Sweden; 2 Centre for Person-Centred Care (GPCC), Institute of Health and Care Sciences, Sahlgrenska Academy, University of Gothenburg, Gothenburg, Sweden; 3 Reumatikerfӧrbundet, Stockholm, Sweden; 4 Psychometric Laboratory for Health Sciences, Faculty of Medicine and Health, University of Leeds, Leeds, UK; 5 Department of Rehabilitation and Movement Science, University of Vermont, Burlington, Vermont, USA; 6 Swiss Paraplegic Research, Nottwil, Switzerland; 7 Department of Physical Medicine and Rehabilitation, Faculty of Medicine, Ankara University, Ankara, Turkey; 8 Department of Biostatistics, Faculty of Medicine, Ankara University, Ankara, Turkey; 9 Department of Health Sciences and Health Policy, University of Lucerne, Lucerne, Switzerland; 10 Leeds Institute of Rheumatic and Musculoskeletal Medicine, University of Leeds, and NIHR Leeds Biomedical Research Centre, Leeds Teaching Hospitals NHS Trust, Leeds, UK

**Keywords:** osteoarthritis, questionnaire, patient, reported outcome measure

## Abstract

**Introduction:**

Patient-reported outcome measures (PROM) or self-completed questionnaires have been used to report outcomes in osteoarthritis (OA) for over 35 years. Choices will always need to be made about what should be measured and, if relevant, what would be the most appropriate PROM to use. The current study aims to describe the available PROMs used in OA and their performance quality, so that informed choices can be made about the most appropriate PROM for a particular task.

**Methods:**

The study included a systematic search for PROMs that have been in use over 17 years (period 2000–2016), and to catalogue their psychometric properties, and to present the evidence in a user-friendly fashion.

**Results:**

78 PROMs were identified with psychometric evidence available. The domains of pain, self-care, mobility and work dominated, whereas domains such as cleaning and laundry and leisure, together with psychological and contextual factors, were poorly served. The most frequently used PROMs included the Western Ontario McMaster Osteoarthritis Index, the Short Form 36 and the Knee Disability and Osteoarthritis Outcome Score which, between them, appeared in more than 4000 papers. Most domains had at least one PROM with the highest level of psychometric evidence.

**Conclusion:**

A broad range of PROMs are available for measuring OA outcomes. Some have good psychometric evidence, others not so. Some important psychological areas such as self-efficacy were poorly served. The study provides a current baseline for what is available, and identifies the shortfall in key domains if the full biopsychosocial model is to be explored.

**Key messages**

**What is already known about this subject?**

- Patient-reported outcome measures (PROM) are commonly used for osteoarthritis (OA) outcomes.
- Some PROMs, for example, Western Ontario McMaster Osteoarthritis Index, are well known.

**What does this study add?**

- This is the first comprehensive review of all PROMs used in published OA studies, including trials.
- This work provides a detailed analysis of the psychometric properties of all OA PROMs and highlights shortfalls in PROMS measuring several domains.

**How might this impact on clinical practice?**

- This work provides a wide range of outcomes for domains that may not usually be considered by clinicians.
- Clinicians can make choices about tools based on their published robustness.

## Introduction

**Figure 1:**
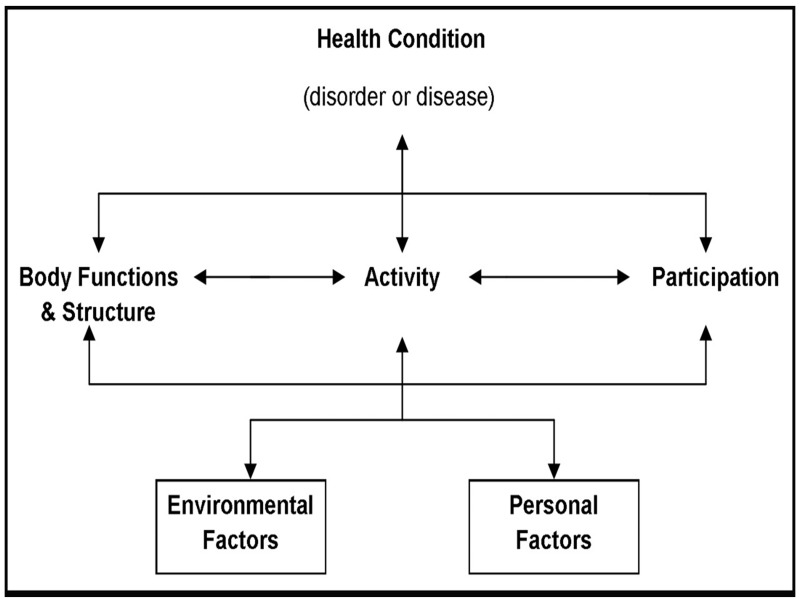
The integrative biopsychosocial model of the International Classification of Functioning, Disability and Health (ICF)^4^

Studies on the lived experience of those with osteoarthritis (OA) show that most facets of life can be affected by this long-term condition.^1–3^ As a result, every aspect of the biopsychosocial model becomes a potential outcome measure for the routine monitoring of patients’ progress, for clinical trials and for epidemiological studies.^4^ Here an ‘outcome’ is defined as any indicator (variable) which is used to detect change in health status or quality of life as part of routine clinical care, or a variety of interventional studies or, for example, longitudinal epidemiological studies.^5 6^ While biomedical indicators can be used from time to time, for those with long-term conditions, many outcomes will consist of simple questionnaires measuring one or more aspects of the conditions’ impact as perceived by the persons themselves. Consequently, the patient-reported outcome measure (PROM), or self-completed questionnaire, has been used to report outcome for over 35 years.^7^ Here we define PROM as any patient (or proxy) completed questionnaire where a set of items are summated to give a total score, or a series of subscale scores, or both. Domains common in OA would include pain, physical functioning and quality of life, and most can be linked to the International Classification of Functioning, Disability and Health (ICF) which has been prescribed as the basis of health recording in eHealth Informatics.^4^ Thus, the rheumatologist and other health professionals associated with intervention and research will be familiar with a range of outcomes ranging from joint destruction (eg, ICF- s73021: joints of hands and fingers), pain (b280: sensation of pain), sleep (b134: sleep functions), mobility (d4), self-care (d5) and work (eg, d8451—maintaining a job).^8 9^ Together, along with environmental and psychological factors (eg, self-efficacy), they form the familiar biopsychosocial model that defines the patients’ lived experience of OA (figure 1).^10–12^

Whether in the context of a clinical trial, or other intervention study, or to monitor the routine care of the patient, choices will need to be made about what should be measured and, if relevant, what would be the most appropriate PROM to use. Systematic reviews have been made on PROMs to help select the most appropriate PROM with the best evidence, but these are often conditional in some way, for example, joint specific, PROM specific or some particular kind of intervention.^13–15^ Given the vast number of potential outcomes, and the number of PROMs available, together with the ever-developing ‘quality standards’ that define the psychometric attributes of the PROM,^16^ a general update of available PROMs across all relevant domains should be of value. Consequently, the current study, funded under the European League Against Rheumatism (EULAR) PROM programme of work, set out to provide the rheumatology community with a review of available PROMs used in OA, and their quality standards, so that the health professional and/or researcher can make informed choices about the most appropriate PROM for their particular need.

## Methods

The study set out to identify the PROMs that have been in use over 17 years (period 2000–2016), and to systematically catalogue their psychometric properties, and to present the evidence in a user-friendly fashion.

### The systematic search

#### Search strategy

The search strategy was developed by the authors at a consensus meeting in early 2012, and in accordance with the Preferred Reporting Items for Systematic Reviews and Meta-Analyses guidelines.^17^ Electronic searches were performed in databases indexing health-related journals using Medline via PubMed and Scopus. Three different searches were used; the first to identify PROMs in use during the specified period (2000–2016); the second to identify papers for a specific PROM where some form of psychometric evidence was present; the third to count the number of times a PROM was used during the search period. An example of the initial PubMed search criteria is found in online supplementary file 1.

10.1136/rmdopen-2018-000715.supp1 Supplementary data

The second search simply adds the name of the PROM using ‘AND’ as Boolean operator to the first part of the search, but without giving a specified period, as the psychometric evidence could arise from any period following the construction of the PROM. This was to identify the relevant psychometric evidence associated with the PROM. The third search removes the psychometric parameters to simply count the use of the PROM in OA during the period 2000–2016. Targeted hand-searching of reference lists and other supplementary sources, such as textbooks, was also performed.

#### Process of selection and data extraction

Potential papers with a candidate PROM identified in search 1 were then screened by two independent reviewers. This included independent screening of the titles and abstracts. For search 2, having added the name of the PROM to the search criteria, papers were included if they met the following criteria: (1) the subjects related to the evidence had OA and the evidence was (mostly) OA specific; (2) one or more of the chosen psychometric criteria specific to the PROM (or its subscales) in question (eg, reliability) were reported in the article; (3) the article was in English; and (4) it was available in full text. These selected papers were again reviewed by two independent researchers and any disagreements were discussed and resolved with a third reviewer.

### Reporting

The results are reported in a series of hierarchically structured tables: (1) overall summary table—main body; (2) PROM-specific summaries—online supplementary file 2; and (3) detailed evidence—online supplementary file 4 of papers used for evidence. The results are catalogued according to well-known domains such as pain, physical function and quality of life with associated ICF classification following, where relevant, in parentheses. Where a PROM is multidomain (ie, subscales) their evidence is presented at the domain level and at any aggregate level above if total scores are produced. Where a PROM has a total score, and covers more than one domain, they are classified under, for example, ‘physical functioning’ (eg, where the PROM has both self-care and mobility domains). Thus, many well-known PROMs will appear more than once, under subscale-specific evidence and at some level of aggregation. Evidence for validity of a subscale will be accepted at the total PROM level (conditional on it being for OA) as this could, for example, be part of a factor analysis of domain structures. Reliability must be specific to the subscale or aggregate domain, and where several studies report, for example, internal consistency reliability (α), the average of those values will be used to determine the reporting level for reliability.

10.1136/rmdopen-2018-000715.supp2 Supplementary data

10.1136/rmdopen-2018-000715.supp4 Supplementary data

It must be noted that the evidence presented here is condition specific; so while a generic PROM may have considerable evidence of validity in other conditions or in mixed samples, if there is no specific evidence within OA, it will be rated as such. Likewise, it is possible that subscales that have been reported may have no separate psychometric evidence for OA, but a total score may do so. Evidence from adaptations into different languages were accepted if some psychometric evidence was forthcoming. Consequently, for a PROM to be included in this review, psychometric evidence for OA from some aspect of the PROM must be evident.

### Psychometric evidence

An independent full-text review of each paper identified the psychometric evidence. This was collated in accord with the domains of the COnsensus-based Standards for the selection of health Measurement INstruments COSMIN checklist^16^ (see online supplementary file 3 for the papers associated with a given PROM) and summarised according to the Outcome Measures in Rheumatology (OMERACT) filter of truth (validity), discrimination (reliability) and feasibility (see online supplementary file 2 for this level of analysis).^18^ Consequently, evidence is collated which informs on whether the PROM is generic or disease specific, the number of items and their response options, its overall use (counted in PubMed) and reliability (internal consistency, test–retest reliability, intraclass correlation coefficient and measurement error), validity (content, construct, criterion) and feasibility of use.

10.1136/rmdopen-2018-000715.supp3 Supplementary data

**Figure 2:**
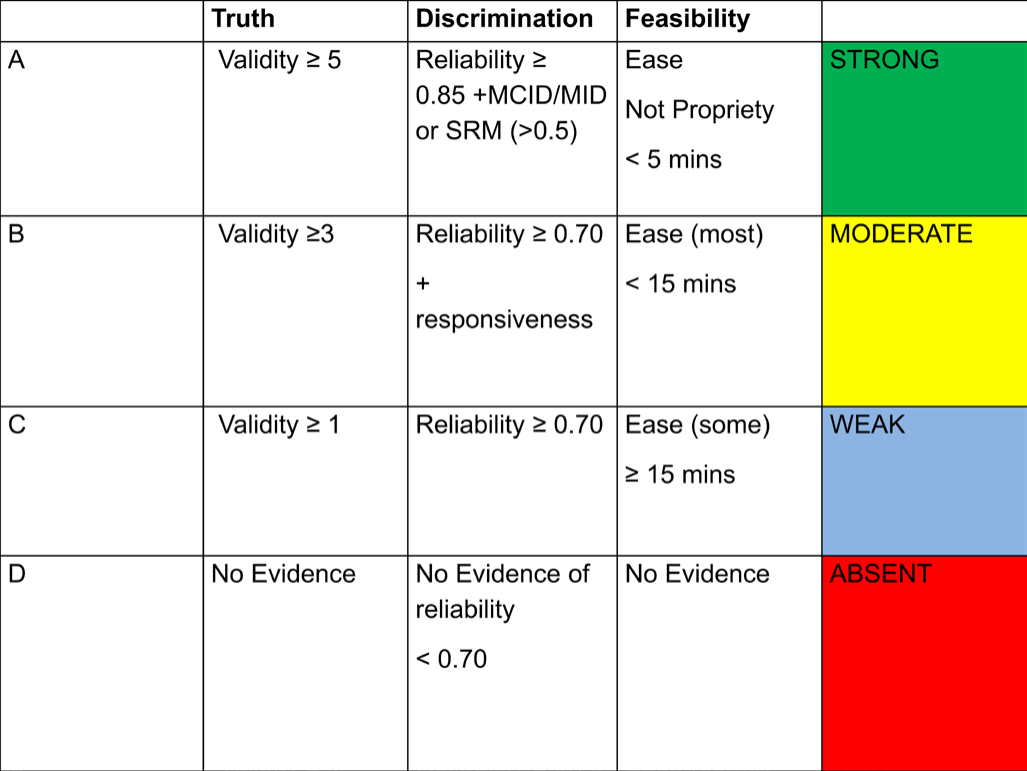
Summary of quality and quantity of reported psychometric evidence of patient-reported outcome measures (PROM) (based on the Outcome Measures in Rheumatology (OMERACT) filter). Validity: quantity of evidence (this must be separate papers providing appropriate supportive evidence). Discrimination: reliability is a requirement, and reflects the degree of discrimination available. MID/MCID and SRM regarded as best quality for responsiveness. Feasibility: understandable and quick to complete from the patient perspective. Availability irrespective of resources.

Discrimination is evaluated by the magnitude of internal consistency reliability, and whether or not some form of Minimally Important Difference (MID)/Minimally Clinical Important Difference (MCID)/Standardised Response Mean(SRM) is presented. For validity, certain PROMs may have been developed originally in another condition (eg, rheumatoid arthritis) or generally for arthritis, and subsequently validated for OA. Where this is the case, and their additional validation is within OA, we designate them as a ‘hybrid’ disease-specific PROM (marked D* in the PROM-specific summary tables in online supplementary file 2). Due to the myriad of ways that validity can be reported, the PROM-specific summary measure focuses on the weight of evidence in support of the validity of the PROM. A negative finding (eg, failure of confirmatory factor analysis) would count as a −1 for support. For feasibility, in the current study the focus is on how easy it is to understand and how quickly the PROM can be completed, as rated by a patient research partner educated in research by the Swedish patient organisation (GP). The partner was asked to rate each PROM according to the ease of completion (1: Impossible to 4: Very easy) and the time for completion (1:<5 min to 4: 31 min or more). This, together with the proprietary status of the PROM, allows for summarising under the feasibility aspect of the OMERACT filter. The summary is presented in a colour-coded format (figure 2). Consequently, a PROM which has more than five separate pieces of evidence of validity, has both reliability and responsiveness evidence at the highest levels, can be completed in less than 5 min with ease and is free for use in all not-for-profit settings will be rated green on all three OMERACT filter parameters, and its summary rating will be green. If on the other hand the PROM was proprietary, then the feasibility rating would be yellow, and so would be the summary rating, which cannot be higher than the lowest rating of any of the three filter categories.

## Results

**Figure 3:**
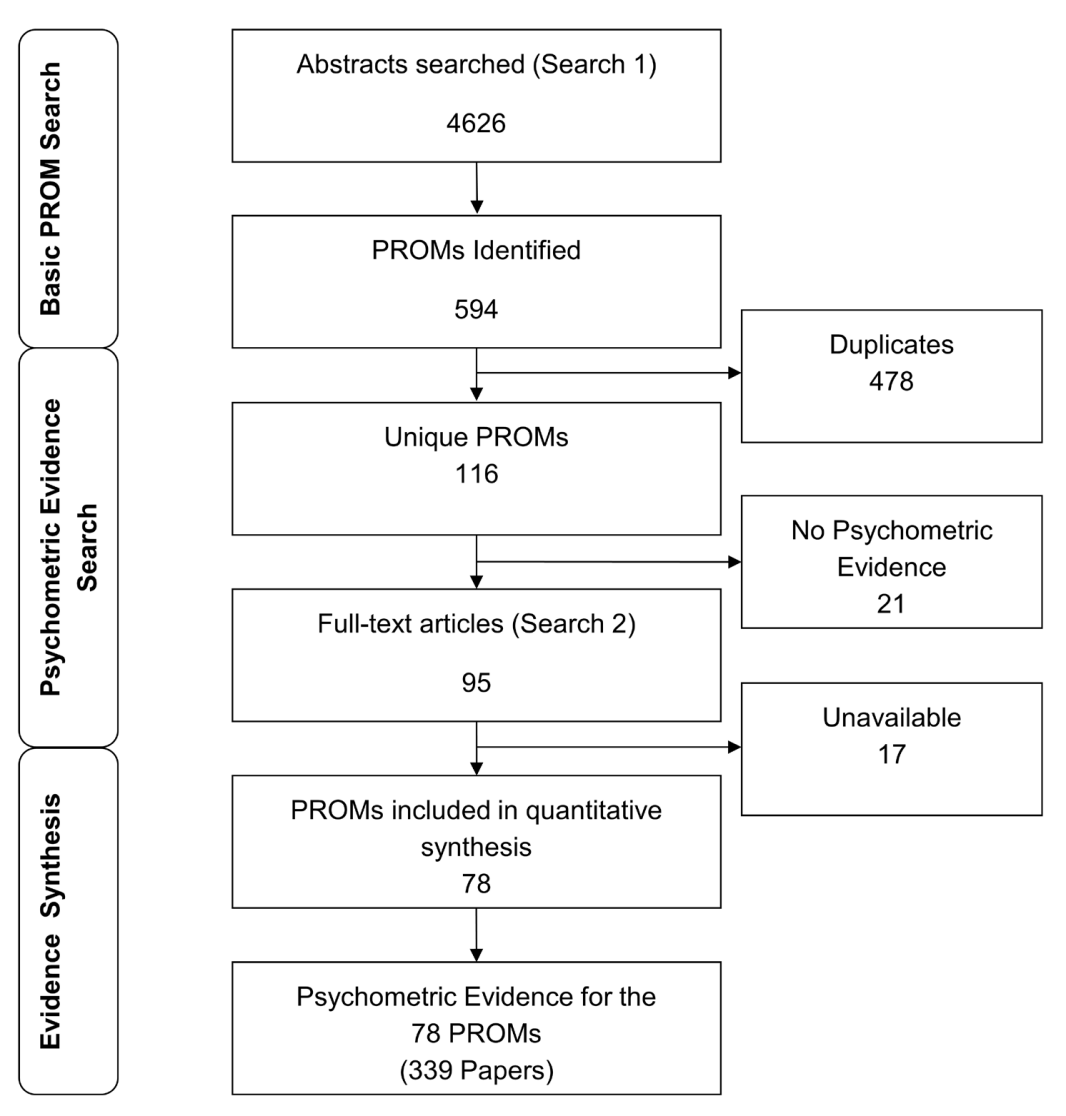
PRISMA flow diagram of search results. PRISMA, Preferred Reporting Items for Systematic Reviews and Meta-Analyses; PROM, patient-reported outcome measure.

Search 1 identified 4626 abstracts with potential PROMs (figure 3). These revealed 595 PROMs, but excluding replications this left 116 unique instruments satisfying the above definition of PROM. No psychometric evidence (specific to OA) was available for 21 of these PROMs, all of which were generic, and often used infrequently. For example, the Epworth Sleep Scale was mentioned in six papers which included OA, but none had psychometric evidence specific to OA. Of the 95 remaining PROMs, 17 were unavailable, leaving 78 PROMs having been used, and with psychometric evidence, and available. For example, the Patient’s Global Assessment of Response to Therapy questionnaire, while psychometric evidence was found, the scale could not be obtained.

Many of these PROMs had subscales, and so were evaluated at both domain and total score level, where appropriate. This gave rise to 157 separate assessments, the overall summary of which can be seen in table 1. Most domains represented in tables 1-14 in online supplementary file 2) had one or more disease-specific PROMs. Some were hybrid (D*), having been developed in another condition, and revalidated for OA.

**Table 1:** Summary of overall evidence for PROMs

Domain(s)	PROMs (n) All/disease specific		Strong (Green)	Moderate (Yellow)	Weak (Blue)	Absent (Red)	ICF	Notes	Detailed reporting Tables in online supplementary file 2
Emotional functions and mental health	12	5	0	4	6	2	b152		1
Pain	24	12	3	7	11	3	b280		2
Stiffness	5	4	2	1	2	0	b780		3
Other symptoms	7	0	0	1	5	1	b134 b144 b210 b4552	Sleep cognition Seeing fatigue	4
Mobility	18	8	2	3	11	2	d4		5
Self-care	8	6	2	1	4	1	d5		6
Domestic	1	1	0	0	1	0	D6		7
Work	13	3	1	0	12	0	d845		8
Social functioning	12	6	0	1	8	3	d7 d8	Social functioning Recreation Lifestyle	9
Physical functioning	26	7	5	3	10	8	D4-d6		10
Physical and social functioning	13	2	1	2	8	2	d4-d9		11
Other psychological and social and societal support	13	2	0	1	5	7	Personal and environmental factors	Social support Satisfaction with services Self-efficacy	12
Quality of life, well-being	5	2	1	1	3	0			13
Health utilities	5	0	0	2	2	1			14
Total	152	58	17	27	88	30			

ICF, International Classification of Functioning, Disability and Health; PROM, patient-reported outcome measure.

A total of 339 papers were reviewed to ascertain the psychometric evidence (some had more than one PROM, and would appear multiple times). Pain, mobility, self-care and work dominated the measured domains, with physical functioning a major complex domain (in that it measures two or more underlying domains such as self-care and mobility). All these domains have a range of PROMs satisfying both ‘good’ and ‘moderate’ criteria on the OMERACT filter, including disease-specific instruments. Work, while well represented, had fewer PROMs rated as ‘good’ or ‘moderate’. In contrast, domains associated with instrumental activities of daily living such as cleaning or laundry, or for other aspects of participation such as leisure activities, were poorly served by available PROMs, as were psychological aspects such as ‘Self-efficacy’, and relevant environmental factors.

The most frequently used PROMs are shown in table 2, dominated by the Western Ontario McMaster Osteoarthritis Index, the Short Form 36 and the Knee Disability and Osteoarthritis Outcome Score which, between them, appeared in more than 4000 papers, and more use during the search period than the remaining seven listed PROMs. Domain-specific assessments are found in tables 1–14 in online supplementary file 2. Where the domain-specific evidence is obtained from a subscale, this is indicated as such within parentheses. Otherwise the PROM will have a designation of ‘Total’ to indicate the evidence arises from the total score. In these tables, ‘Use’ represents the number of identified studies reporting having used the PROM in patients with OA. The PROM-specific references are to be found in online supplementary file 3, and the papers contributing to the detailed psychometric evidence are to be found in online supplementary file 4, catalogued in the same order as the PROM-specific references.

**Table 2:** Ten most frequently used PROMs in osteoarthritis published papers: 2000–2016

No	Name	Acronym	Reference (Supp 3)
1	Western Ontario McMaster Osteoarthritis Index	WOMAC	28
2	Medical Outcome Studies Short Form 36	SF-36	20
3	Knee Disability and Osteoarthritis Outcome Score	KOOS	15
4	Oxford Knee Score	OKS	54
**5**	Disabilities of the Arm, Shoulder and Hand	DASH	39
6	EUROQoL	EQ5-D	66
7	Medical Outcomes Study Short Form 12-Item	SF-12	55
8	Hip Disability and Osteoarthritis Outcome Score	HOOS	54
9	Pain Catastrophizing PROM	PCS	23
10	Oxford Hip Score	OHS	53

Supp 3: reference number in online supplementary file 3.

PROM, patient-reported outcome measure.

## Discussion

Seventy-eight PROMs with some level of supporting psychometric evidence, available and assessed by the patient partner were catalogued according to a variety of commonly used domains. Found in published papers between 2000 and 2016, almost all domains had at least one PROM rated as ‘good’ or ‘moderate’ (green or yellow) on the OMERACT filter summary. The domains of pain (ICF-b280), mobility (d4) and self-care (d5) were dominant, along with work (d8451). This is unsurprising as these are those aspects of OA that are commonly reported, and include potentially modifying factors relevant for intervention.^19 20^ As such, these domains represent good candidates for inclusion in clinical trials and routine clinical monitoring, along with quality of life which is also considered important from a ‘whole person’ perspective.^21 22^

While some well-known PROMs appear to have no evidence of their reliability and/or validity, there are a number of reasons for this. For example, the original Arthritis Impact Measurement Scale (AIMS) paper involved 104 cases from a mixed rheumatology clinic sample, 31% of which had OA.^7^ The psychometric results were not disaggregated to the condition-specific level. Furthermore, sometimes PROMs with various subdomains only have evidence at the total summed score level, across all domains, so the individual domains would indicate no evidence for reliability. Likewise, caution must also be taken with more recently developed PROMs which may not yet have accumulated sufficient evidence for validity to warrant a green indicator (eg, Measure of Intermittent and Constant Osteoarthritis Pain).

On balance, any PROM that has a yellow or green indicator will be worth considering, conditional on the year of publication. Yellow may indicate a propriety status if the feasibility indicator is also yellow. As PROMs appear to be treated as a commodity by certain large-scale test companies, where the (partial) rights to the PROM are bought from the original developers, and subsequently made propriety, it will be essential to check the status of any PROM to ascertain its current propriety status. Readers are also encouraged to view the relevant published papers listed in online supplementary file 3 and, if required, the detailed psychometric evidence on a PROM-specific spreadsheet (available from the lead author). As new evidence is emerging continuously, having chosen a potential PROM, a quick search to update (post-2016) the existing evidence would be wise, particularly if existing evidence appears weak in the current search, and/or the scale is relatively new.

The lack of adequate PROMs used to ascertain aspects of participation such as leisure, together with psychological and environmental factors, is of concern, as this limits the available mediators and moderators for analysis of the more complete biopsychosocial model. For example, treatment focused on self-efficacy and psychological distress has been reported as most effective in improving the quality of well-being of those with OA aged 60 years and over.^22^ Also it has been argued that approaches aimed at enhancing social functioning (reducing participation restriction) for those with OA should consider decreasing environmental barriers.^23^ Furthermore, although ‘work’ is well represented, other aspects of participation such as leisure, economic life and ‘religion and spirituality’ are absent, all of which may be important to understand cultural differences in coping with OA.^24 25^ While it is not uncommon to have individual items addressing these issues as part of measuring a wider domain, some thought should be given to whether or not they have sufficient importance to warrant a specific domain in any future PROM development. Finally, it is also of concern that about 20% of the PROMs used during the period had no supporting psychometric evidence for their use in OA.

The information presented here arises from just one of several EULAR-funded initiatives to catalogue the available PROMs across several rheumatic diseases, leading to the EULAR Outcome Measures Library.^26^ There were several limitations to the current study. Some difficulty was encountered with subscale-specific judgement of time taken and ease of interpretation. Generally, PROMs are not given to patients at the subscale level, and so the judgement is always based on the full PROM from the patient perspective. Only the reliability evidence was subscale specific. Similarly, validity is judged by the whole PROM, as sometimes the evidence about the structure of a domain is forthcoming for a confirmatory analysis of the whole PROM. Furthermore, no attempt was made to judge the quality of the evidence presented, just the weight of evidence in support of the PROM in the case of truth (validity). The assumption underlying this is that the greater the level of psychometric evidence, the more likely that some will be of good quality. Finally, the feasibility was judged by just one person who, as specifically trained in research, may have had more experience of PROMs than is normal, so that judgement may be more generous to the PROM than may be the case otherwise.

Given the age-related prevalence of OA, and the ageing of the population in western societies, maintaining health status and quality of life among those with OA is likely to be a major public health issue. Currently, a rich catalogue of PROMs is available for measuring these domains, although some potentially important domains such as leisure and ‘self-efficacy’ are poorly served. Rheumatologists and other allied health professionals working with those with OA will need to keep in mind the wider biopsychosocial model, together with recent developments in the area of e-health informatics which prescribes the ICF as the basis of reporting health, defined as functioning.^27^
