# Supplementary material for: Patient-reported outcome measures in osteoarthritis: a systematic search and review of their use and psychometric properties
Source: RMD Open. 2018 Dec 16;4(2):e000715. doi: 10.1136/rmdopen-2018-000715 (PMC6307597; doi:10.1136/rmdopen-2018-000715)
Supplement: Supplementary data [file rmdopen-2018-000715supp002.pdf]

Supplementary File 2

Summary of PROM-Specific Evidence

Supplementary Table 1. General characteristics and Summary Psychometric Review of all versions of identified PROMs:

***Emotional Functions and mental Health* (b126, b152)**

Name (Domain)	Acronym	First Author/ Year	REF (A)	Generic/ Disease Specific	N of items	Response Options	Recall period	Use	Psychometric Summary Truth:	Discrimination:	Feasibility:	Overall
Arthritis Impact Measurement PROMs 2 (Tension)	**AIMS2**	Meenan R 1992.	1	D*	5	5	Past month	60				
Arthritis Impact Measurement PROMs 2 (Mood)	**AIMS2**	Meenan R. 1992	1	D*	5	5	Past month	60				
Arthritis Impact Measurement PROMs 2-SF (Affect)	**AIMS2-SF**	Guillemin F. 1997	2	D*	4	5	Past month	41				

Name (Domain)	Acronym	First Author/ Year	REF (A)	Generic/ Disease Specific	N of items	Response Options	Recall period	Use	Psychometric Summary Truth:	Discrimination:	Feasibility:	Overall
Center for Epidemiologic Studies Depression PROM (Mental functions: Depression)	**CES-D**	Radloff LS. 1977	4	G	20	4	Past week	32				
General Well-Being Index (Mental functions: Psychological distress)	**GWBI**	Gaston JE. 2005	5	G	22	5	Past 2 weeks	1				
Hospital Anxiety and Depression (anxiety and depression)	**HADS**	Zigmond AS. 1983	6	G	14	4	Past week	47				
Osteoarthritis of Knee Hip Quality of Life (Mental Health)	**OAKHQOL**	Rat A-C. 2005	7	D	13	0-10	?	14				
Medical Outcomes Study Short Form 36-Item (Emotional wellbeing)	**SF-36**	Ware JE. 1992	20	G	5	6	Past 4 weeks	989				

Name (Domain)	Acronym	First Author/ Year	REF (A)	Generic/ Disease Specific	N of items	Response Options	Recall period	Use	Psychometric Summary Truth:	Discrimination:	Feasibility:	Overall
PROMIS Anxiety SF (Total)	**PROMIS-ANX-SF**	Pilkonis PA 2011	77	G	7	5	Past Week	1				
PROMIS Depression SF (Total)	**PROMIS-DEP-SF**	Choi SW 2010	75	G	8	5	Past Week	1				
Western Ontario Osteoarthritis of the Shoulder Index (Emotional Function)	**WOOS**	Lo I. 2001	8	D	3	0-100	Past week	34				
Western Ontario Shoulder Instability Index (Emotional Function)	**WOSI**	Kirkley A. 1998	9	G	3	0-100	Past week	10				

Evidence: Strong; Moderate; Weak; Absent

Supplementary Table 2. General characteristics and Summary Psychometric Review of all versions of identified PROMs: *Pain* (b280)

**Name (Domain)**	Acronym / Name	**First Author**	**REF**	**Generic/ Disease Specific**	**N of items**	**Response Options**	**Recall period**	**Use**	**Psychometric Summary** Truth:	Discrimination:	Feasibility:	**Overall**
Arthritis Impact Measurement PROMs (Arthritis Pain)	**AIMS**	Meenan R. 1980	10	G	4	6	Past month	28				
Arthritis Impact Measurement PROMs 2 (Arthritis Pain)	**AIMS2**	Meenan R. 1992	1	D*	5	5	Past month	34				
Arthritis Impact Measurement PROMs 2-SF (Symptoms: Pain and stiffness)	**AIMS2-SF**	Guillemin F. 1997	2	D*	3	5	Past 4 weeks	20				
Australian/Canadian Hand Osteoarthritis Index (Pain)	**AUSCAN**	Bellamy N. 2002	11	D	5	5<br>100 (VAS)	48 hours	89				
Brief Pain Inventory Long/Short version (Pain)	**BPI**	Cleeland CS 1994	12	G	20/11	0-10	24 hours	86				
Foot Function Index (Pain)	**FFI**	Budiman-Mak E. 1991	13	G	9	1-10	Past week	36				

Name (Domain)	Acronym / Name	First Author	REF	Generic/ Disease Specific	N of items	Response Options	Recall period	Use	Psychometric Summary Truth:	Discrimination:	Feasibility:	Overall
Hip Disability and Osteoarthritis Outcome Score (Pain)	**HOOS**	Klässbo M. 2003	14	D	9	5	Last week	48				
Knee Injury and Osteoarthritis Outcome Score (Pain)	**KOOS**	Roos EM. 1998	15	D	9	5	Last week	886				
Lequesne (Algofunctional) Index of hip/knee (Pain)	**LAI**	Lequesne M. 1997	16	D	5	9	Present	32				
Lequesne Index of Severity for Osteoarthritis of the Hip (Pain)	**LISOH**	Lequesne M. 1987	17	D	5	9	Present	18				
MGill Pain Questionnaire (Total)	**MPQ**	Melzack R. 1975	18	G	20	Varies Words	present	31				
Measure of Intermittent and Constant Osteoarthritis Pain (Total)	**ICOAP**	Hawker G. 2008	19	D	11	5	Past week	28				

Name (Domain)	Acronym / Name	First Author	REF	Generic/ Disease Specific	N of items	Response Options	Recall period	Use	Psychometric Summary<br>Truth:	Discrimination:	Feasibility:	Overall
Medical Outcomes Study Short Form 36-Item (Bodily Pain)	**SF-36**	Ware JE. 1992	20	G	2	5/6	Past 4 weeks	989				
Nottingham Health Profile (Pain)	**NHP**	Hunt SM 1985	21	G	8	0-1	Present	38				
Oxford Elbow Score (Pain)	**OES**	Dawson J. 2008	22	G	4	5	Past 4 weeks	2				
Osteoarthritis of Knee Hip Quality of Life (Pain)	**OAKHQOL**	Rat A-C. 2005	7	D	4	0-10	?	14				
Pain Catastrophizing PROM (Total	**PCS**	Sullivan MJ. 1995	23	G	13	5	when in pain	126				
Patient Rated Elbow Evaluation (Total)	**PREE**	MacDermid JC 2001.	24	G	5	11	Past week	1				
Patient Rated Wrist Evaluation (Pain)	**PRWE**	MacDermid JC 1996.	25	G	5	11	Past week	18				

Name (Domain)	Acronym / Name	First Author	REF	Generic/ Disease Specific	N of items	Response Options	Recall period	Use	Psychometric Summary Truth:	Discrimination:	Feasibility:	Overall
PROMIS Pain Interference - Short Forms (Pain)	**PROMIS-Pain-SF**	Amtmann DA 2010	74	G	6	5	Past Week	2				
Score for assessment and quantification of chronic rheumatic affections of the hand (Pain)	**SACRAH**	Leeb B. 2003	26	G	4	VAS	48 hours	11				
Shoulder Pain and Disability Index (Pain)	**SPADI**	Roach KE. 1991	27	D*	5	VAS/11	Past week	13				
Western Ontario Osteoarthritis of the Shoulder Index (Physical Symptoms)	**WOOS**	Lo I. 2001	8	D	6	VAS	Past week	34				
Western Ontario McMaster Osteoarthritis Index (Pain)	**WOMAC**	Bellamy N. 1988	28	D	17	5, 11, VAS	Currently	2422				

Evidence: Strong; Moderate; Weak; Absent

Supplementary Table 3. General characteristics and Summary Psychometric Review of all versions of identified PROMs: *Stiffness* (Impairment of function – b780)

Name (Domain)	Acronym / Name	First Author	REF	Generic/ Disease Specific	N of items	Response Options	Recall period	Use	Psychometric Summary Truth:	Discrimination:	Feasibility:	Overall
Arthritis Impact Measurement Scales2-SF (Symptoms: Pain and stiffness)	**AIMS2-SF**	Guillemin F. 1997	2	D*	3	5	Past 4 weeks	41				
Hip Disability and Osteoarthritis Outcome Score (stiffness)	**HOOS**	Klässbo M. 2003	14	D	2	5	Last week	130				
Knee Disability and Osteoarthritis Outcome Score (stiffness)	**KOOS**	Roos EM. 1998	15	D	2	5	Last week	886				
Score for assessment and quantification of chronic rheumatic affections of the hand (Stiffness)	**SACRAH**	Leeb B. 2003	26	G	2	0-100	48 hours	11				

Name (Domain)	Acronym / Name	First Author	REF	Generic/ Disease Specific	N of items	Response Options	Recall period	Use	Psychometric Summary Truth: Discrimination: Feasibility:				Overall
Western Ontario McMaster Osteoarthritis Index (Stiffness)	**WOMAC**	Bellamy N. 1988	28	D	2	5, 0-10, 0-100	Currently	2422					

Evidence: Strong; Moderate; Weak; Absent

Supplementary Table 4. General characteristics and Summary Psychometric Review of all versions of identified PROMs:

Other Symptoms (Impairments), including Fatigue

Name (Domain)	Acronym / Name	First Author	REF	Generic/ Disease Specific	N of items	Response Options	Recall period	Use	Psychometric Summary Truth:	Discrimination:	Feasibility:	Overall
Assessment of Quality of Life (physical senses: seeing, hearing, communication)	**AQoL**	Hawthorne G. 1999	3	G	3	4	Last week	13				
Chronic Pain Sleep Inventory (Total)	**CPSI**	Kosinski M. 2007	29	G	5	0-100	Unknown	3				
Knee Outcome Survey: Activities of Daily Living PROM (Mixed Impairments)	**KOS-ADLS**	Irrgang JJ 1998	72	G	6	6	Unknown	10				
Nottingham Health Profile (Sleep)	**NHP**	Hunt SM 1985	21	G	5	0-1	Present	38				
Medical Outcomes Study Short Form 36-Item (Vitality)	**SF-36**	Ware JE. 1992	20	G	4	6	Past 4 weeks	989				
Nottingham Health Profile (Energy)	**NHP**	Hunt SM 1985	21	G	3	0-1	Present	38				

Name (Domain)	Acronym / Name	First Author	REF	Generic/ Disease Specific	N of items	Response Options	Recall period	Use	Psychometric Summary Truth: Discrimination: Feasibility:				Overall
World Health Organisation Disability Assessment Schedule –II (Cognition)	**WHODAS-II**	WHO 2004	30	G	6	5	Last 30 days	1					

Evidence: Strong; Moderate; Weak; Absent

Supplementary Table 5. General characteristics and Summary Psychometric Review of all versions of identified PROMs:– *Mobility* (Activities and participation- d4)

Name (Domain)	Acronym / Name	First Author	REF	Generic/ Disease Specific	N of items	Response Options	Recall period	Use	Psychometric Summary Truth:	Discrimination:	Feasibility:	Overall
Arthritis Impact Measurement Scale2 (Mobility)	**AIMS2**	Meenan R. 1992	1	D*	5	5	Past month	60				
Arthritis Impact Measurement Scales 2 (Walking and bending)	**AIMS2**	Meenan R. 1992	1	D*	5	5	Past month	60				
Arthritis Impact Measurement Scales 2 (Hand and finger)	**AIMS2**	Meenan R. 1992	1	D*	5	5	Past month	60				
Arthritis Impact Measurement Scales 2 (Arm function)	**AIMS2**	Meenan R. 1992	1	D*	5	5	Past month	60				
Arthritis Impact Measurement Scale 2-SF (upper and lower extremity function)	**AIMS2-SF**	Guillemin F. 1997	2	D*	26	5	Past 4 weeks	41				

Name (Domain)	Acronym / Name	First Author	REF	Generic/ Disease Specific	N of items	Response Options	Recall period	Use	Psychometric Summary Truth:	Discrimination:	Feasibility:	Overall
Australian/Canadian Hand Osteoarthritis Index (Function)	**AUSCAN**	Bellamy N. 2002	11	D	9	5	48 hours	89				
Baecke Physical Activity Questionnaire (sports)	**BQ**	Baecke J. 1982	31	G	10	5	Not specified	1				
Baecke Physical Activity Questionnaire (leisure)	**BQ**	Baecke J. 1982	31	G	4	5	Not specified	1				
Cochin Hand Function PROM	**CHFS**	Duruöz M. 1996	32	G	18	5	Not specified	22				
Hip Disability and Osteoarthritis Outcome Score (sports and recreation)	**HOOS**	Klässbo M. 2003	14	D	4	5	Last week	130				
Knee Disability and Osteoarthritis Outcome Score (Sports and recreation)	**KOOS**	Roos EM. 1998	15	D	5	5	Last week	886				

Name (Domain)	Acronym / Name	First Author	REF	Generic/ Disease Specific	N of items	Response Options	Recall period	Use	Psychometric Summary Truth:	Discrimination:	Feasibility:	Overall
Knee Outcome Survey: Activities of Daily Living Scale (Mixed Impairments)	**KOS-ADLS**	Irrgang JJ 1998	72	G	8	6	Unknown	10				
Late-Life Function and Disability Instrument (Function)	**LLFDI**	Haley SM. 2002	38	G	32 +8 if using walking devise	5	On a typical day	8				
Lower Extremity Activity Scale (Total)	**LEAS**	Saleh KJ. 2005	33	G	1	18	Regular daily activity	14				
Mayo Hip Score (Total)	**MHS**	Sing JA 2016	71	G	7	3-6	Unknown	8				
Walking Impairment Questionnaire (Total: mobility)	**WIQ**	Regensteiner J. 1990	35	G	14	5	Past week	2				
Western Ontario McMaster Osteoarthritis Index (Mobility - functions)	**WOMAC**	Bellamy N. 1988	28	D	17	5, 0-10, 0-100	Currently	2422				

Name<br>(Domain)	Acronym<br>/ Name	First Author	REF	Generic/<br>Disease Specific	N<br>of items	Response<br>Options	Recall<br>period	Use	Psychometric Summary<br>Truth:	Discrimination:	Feasibility:	Overall
World Health Organisation Disability Assessment Schedule –II<br>(Mobility)	**WHODAS-II**	WHO<br>2004	30	G	5	5	Last 30 days	1				

Evidence: Strong; Moderate; Weak; Absent

Supplementary Table 6. General characteristics and Summary Psychometric Review of all versions of identified PROMs:

***Self Care* (Activities and participation – d5)**

**Name (Domain)**	**Acronym / Name**	**First Author**	**REF**	**Generic/ Disease Specific**	**N of items**	**Response Options**	**Recall period**	**Use**	**Psychometric Summary Truth:**	**Discrimination:**	**Feasibility:**	**Overall**
Arthritis Impact Measurement Scales 2 (Self care)	**AIMS2**	Meenan R. 1992	1	D*	4	5	Past month	60				
Assessment of Quality of Life (self care)	**AQoL**	Hawthorne G. 1998	3	G	3	4	Last week	13				
Australian/Canadian Hand Osteoarthritis Index (Function: self care and domestic)	**AUSCAN**	Bellamy N. 2002	11	D	9	5<br>100 (VAS)	48 hours	89				
Cochin Hand Function PROM (self care)	**CHFS**	Duruöz M. 1996	32	D*	18	6	NA	2				
Functional Index for Hand Osteoarthritis (Total)	**FIHOA**	Dreiser RL 1995	72	D	10	4	N/A	24				
Hip Disability and Osteoarthritis Outcome Score (ADL)	**HOOS**	Klässbo M 2003	14	D	17	5	Last week	130				

Name (Domain)	Acronym / Name	First Author	REF	Generic/ Disease Specific	N of items	Response Options	Recall period	Use	Psychometric Summary Truth:	Discrimination:	Feasibility:	Overall
Knee Disability and Osteoarthritis Outcome Score (function in daily living (ADL))	**KOOS**	Roos EM. 1998	15	D	17	5	Last week	886				
World Health Organisation Disability Assessment Schedule –II (Self-care)	**WHODAS-II**	WHO 2004	30	G	4	5	Last 30 days	1				

Evidence: Strong; Moderate; Weak; Absent

Supplementary Table 7. General characteristics and Summary Psychometric Review of all versions of identified PROMs: ***Domestic*** **(Activities and participation – d6)**

Name (Domain)	Acronym / Name	First Author	REF	Generic/ Disease Specific	N of items	Response Options	Recall period	Use	Psychometric Summary Truth:	Discrimination:	Feasibility:	Overall
Arthritis Impact Measurement Scales 2 (Household)	**AIMS2**	Meenan R. 1992	1	D*	4	5	Past month	60				

Evidence: Strong; Moderate; Weak; Absent

Supplementary Table 8. General characteristics and Summary Psychometric Review of all versions of identified PROMs: *Work* (Activities and participation – d845)

Name (Domain)	Acronym / Name	First Author	REF	Generic/ Disease Specific	N of items	Response Options	Recall period	Use	Psychometric Summary Truth: Discrimination: Feasibility:			Overall
Arthritis Impact Measurement Scales 2 (Work)	**AIMS2**	Meenan R. 1992	1	D*	5	5	Past month	60				
Arthritis Work Spillover (Total)	**AWS**	Gignac MA 2006	69	G	6	5	Not Specified	3				
Baecke Physical Activity Questionnaire (work)	**BQ**	Baecke J. 1982	31	G	8	5	Not specified	1				
Chronic Illness Job Strain Scale (Total)	**CIJSS**	Gignac MA. 2007	34	G	13	5	Not specified	1				
Rheumatoid Arthritis Work Instability Scale (Total)	**RA-WIS**	Gilworth G. 2003	36	D*	23	2	At the moment	5				

Name (Domain)	Acronym / Name	First Author	REF	Generic/ Disease Specific	N of items	Response Options	Recall period	Use	Psychometric Summary Truth:	Discrimination:	Feasibility:	Overall
World Health Organisation Disability Assessment Schedule –II (life activities) ICF: domestic work and paid work	**WHODAS-II**	WHO 2004	30	G	4/8	5	Last 30 days	1				
Work Limitations Questionnaire (Total)	**WLQ**	Lerner D. 2001	37	G	25	5	Past 2 weeks	5				
Work Limitations Questionnaire (physical demands)	**WLQ**	Lerner D. 2001	37	G	6	5	Past 2 weeks	5				
Work Limitations Questionnaire (time management)	**WLQ**	Lerner D. 2001	37	G	5	5	Past 2 weeks	5				
Work Limitations Questionnaire (mental-interpersonal demands)	**WLQ**	Lerner D. 2001	37	G	9	5	Past 2 weeks	5				
Work Limitations Questionnaire (output demands)	**WLQ**	Lerner D. 2001	37	G	5	5	Past 2 weeks	5				

Name (Domain)	Acronym / Name	First Author	REF	Generic/ Disease Specific	N of items	Response Options	Recall period	Use	Psychometric Summary Truth:	Discrimination:	Feasibility:	Overall
Workplace Activities Limitations Scale (Total)	**WALS**	Gignac MA. 2005	38	D*	12	5	Not specified	6				
work productivity and activity instrument (Work productivty loss (overall work impairment / absenteeism plus presenteeism)	**WPAI**	Reilly MC 1993	73	G	6	varied	Seven days	9				

Evidence: Strong; Moderate; Weak; Absent

Supplementary Table 9. General characteristics and Summary Psychometric Review of all versions of identified PROMs:

***Social Functioning* (Other Participation)**

Name (Domain)	Acronym / Name	First Author	REF	Generic/ Disease Specific	N of items	Response Options	Recall period	Use	Psychometric Summary Truth:	Discrimination:	Feasibility:	Overall
Arthritis Impact Measurement Scales 2 (Social activity)	**AIMS2**	Meenan R. 1992	1	D*	5	5	Past month	60				
Arthritis Impact Measurement Scales 2-SF (Social interaction)	**AIMS2-SF**	Guillemin F. 1997	2	D*	?	5	Past 4 weeks	41				
Arthritis Impact MeasurementScales 2-SF (Role)	**AIMS2-SF**	Guillemin F. 1997	2	D*	?	5	Past 4 weeks	41				
Assessment of Quality of Life (independent living)	**AQoL**	Hawthorne G. 1999	3	G	?	4	Last week	13				
Assessment of Quality of Life (social relationships)	**AQoL**	Hawthorne G. 1999	3	G	?	4	Last week	13				
Late-Life Function and Disability Instrument (Disability - Participation)	**LLFDI**	Jette AM. 2002	39	G	16	5	At this time in your life	8				

Name (Domain)	Acronym / Name	First Author	REF	Generic/ Disease Specific	N of items	Response Options	Recall period	Use	Psychometric Summary Truth: Discrimination: Feasibility:				Overall
Osteoarthritis of Knee Hip Quality of Life (Social Activities)	**OAKHQOL**	Rat A-C. 2005	7	D	3	0-10	Unknown	14					
Medical Outcomes Study Short Form 36-Item (Social functioning)	**SF-36**	Ware JE. 1992	20	G	4	0-1	Past 4 weeks	989					
Western Ontario Osteoarthritis of the Shoulder Index (Sports/Recreation/ work)	**WOOS**	Lo I. 2001	8	D	5	0-100	Past week	34					
Western Ontario Osteoarthritis of the Shoulder Index (Lifestyle)	**WOOS**	Lo I. 2001	8	D	5	0-100	Past week	34					
World Health Organisation Disability Assessment Schedule –II (Getting along)	**WHODAS-II**	WHO 2004	30	G	5	5	Last 30 days	1					

Name (Domain)	Acronym / Name	First Author	REF	Generic/ Disease Specific	N of items	Response Options	Recall period	Use	Psychometric Summary Truth: Discrimination: Feasibility:			Overall
World Health Organisation Disability Assessment Schedule –II (participation)	**WHODAS-II**	WHO 2004	30	G	8	5	Last 30 days	1				

Evidence: Strong; Moderate; Weak; Absent

Supplementary Table 10. General characteristics and Summary Psychometric Review of all versions of identified PROMs:
***Physical Functioning* (Activity Limitation- Composite Total Scores)**

Name (Domain)	Acronym / Name	First Author	REF	Generic/ Disease Specific	N of items	Response Options	Recall period	Use	Psychometric Summary Truth:	Discrimination:	Feasibility:	Overall
Arthritis Impact Measurement Scales (Total)	**AIMS**	Meenan R. 1980	10	G	45	5?	Past month	60				
Disabilities of the Arm Shoulder and Hand (Total)	**DASH**	Hudak PL. 1996	40	G	30	5	1 week	252				
Evaluation of Daily Living Questionnaire (Total)	**EDAQ**	Nordenskiöld U	78	G	138	4	Last 2 weeks	1				
Health Assessment Questionnaire (DI) (Total)	**HAQ**	Fries JP. 1980	41	G	20	4	Past week	176				
HOOS physical function short form (Total)	**HOOS-PS**	Davis A. 2008	42	D	5	5	Last week	32				
Human Activity Profile (Total)	**HAP**	Fix AJ. 1988	43	G	94	4	At present	16				
Ibadan Knee/Hip Osteoarthritis Outcome Measure (Activity limitation)	**IKHOAM**	Akinpelu A 2007	44	D	28	5	Not specified	3				

Name (Domain)	Acronym / Name	First Author	REF	Generic/ Disease Specific	N of items	Response Options	Recall period	Use	Psychometric Summary Truth:	Discrimination:	Feasibility:	Overall
KOOS physical function short form (Total)	**KOOS-PS**	Perruccio AV 2008	45	D	7	5	Last week	88				
Knee Outcome Survey Activities of Daily Living PROM (ADL)	**KOS-ADLS**	Irrgang JJ. 1998	46	D	13	6	Not specified	10				
Lequesne (Algofunctional) Index of hip/knee (Total)	**LAI**	Lequesne M. 1997	16	G	11	varying	Not Specified	32				
Lower Extremity Functional PROM (activity limitations)	**LEFS**	Binkley JM. 1999	47	G	20	5	Today	44				
McMaster Toronto Arthritis Patient Preference Disability Questionnaire (disability)	**MACTAR**	Tugwell P. 1987	48	G	5	3	Past 2 weeks	12				
Michigan Hand Questionnaire (Total)	**MHQ**	Chung KC. 1998	49	G	37	5	Past week	10				
Modified Health Assessment Questionnaire (Total)	**MHAQ**	Pincus T. 1983	50	G	8	4	Past week	8				

Name (Domain)	Acronym / Name	First Author	REF	Generic/ Disease Specific	N of items	Response Options	Recall period	Use	Psychometric Summary			Overall
									Truth:	Discrimination:	Feasibility:	
Multidimensional Health Assesssment Questionnaire (Total)	**MDHAQ**	Pincus 2005	51	G	10	4	Past week	10				
Oswestry Disability Index (Total)	**ODI**	Fairbank JC 2000.	52	G	10	6	At the moment	30				
Oxford Hip Score (Total)	**OHS**	Dawson J. 1996	53	D*	12	5	Past 4 weeks	106				
Oxford Knee Score (Total)	**OKS**	Dawson J. 1998	54	D*	12	5	Past 4 weeks	252				
Oxford Shoulder Score (Total)	**OSS**	Dawson J. 1996	55	G	12	5	Past 4 weeks	22				
Osteoarthritis of Knee Hip Quality of Life (Physical activity)	**OAKHQOL**	Rat A-C. 2005	7	D	19	0-10	Unknown	14				
PROMIS – Physical Function Short Form (Total)	**PROMIS-PF-SF**	Hays RD 2013	76	G	20/10 6/4	5	Not Stated	2				
Quick Disabilities of the Arm Shoulder and Hand (Total)	**QDASH**	Beaton DE 2005	56	G	11 +8 optional	1-5	Last week	29				

Name (Domain)	Acronym / Name	First Author	REF	Generic/ Disease Specific	N of items	Response Options	Recall period	Use	Psychometric Summary Truth:	Discrimination:	Feasibility:	Overall
Score for assessment and quantification of chronic rheumatic affextions of the hand (Hand Function)	**SACRAH**	Leeb B. 2003	26	G	17	0-100	48 hours	11				
Medical Outcomes Study Short Form 36-Item (Physical functioning)	**SF-36**	Ware JE. 1992	20	G	10	1-3	Past 4 weeks	989				
Medical Outcomes Study Short Form 12-Item (Physical Component)	**SF-12**	Ware JE. 1996	57	G	6	1-6	Past 4 weeks	210				
Shoulder Pain and Disability Index (Disability)	**SPADI**	Roach KE. 1991	27	G	8	VAS/0-10	Past week	13				

Evidence: Strong; Moderate; Weak; Absent

Supplementary Table 11. General characteristics and Summary Psychometric Review of all versions of identified PROMs: *Physical and Social Functioning*. (Impairment, Activities, Participation. Composite Total Scores)

Name (Domain)	Acronym / Name	First Author	REF	Generic/ Disease Specific	N of items	Response Options	Recall period	Use	Psychometric Summary			Overall
									Truth:	Discrimination:	Feasibility:	
Arthritis Impact Measurement Scales (Total)	**AIMS**	Meenan R. 1980	1o	G	45	5?	Past month	60				
Illness Intrusiveness Rating Scale (activity and participation, general)	**IIRS**	Devins GM. 1984	58	G	13	7	Current life situation	1				
Patient Rated Elbow Evaluation (Total)	**PREE**	MacDermid JC. 2001	24	G	20	0-10	Past week	1				
Patient Rated Wrist Evaluation (Total)	**PRWE**	MacDermid JC. 2003	25	D*	15	0-10	Past week	18				
Perceived Impact Problem of Profile (Total)	**PIPP**	Pallant JF. 2006	59	G	23	6	Current	1				

Name (Domain)	Acronym / Name	First Author	REF	Generic/ Disease Specific	N of items	Response Options	Recall period	Use	Psychometric Summary Truth:	Discrimination:	Feasibility:	Overall
Score for assessment and quantification of chronic rheumatic affextions of the hand (Total: pain/stiffness/hand function)	**SACRAH**	Leeb B. 2003	26	G	23	0-100	48 hours	11				
Medical Outcomes Study Short Form 12-Item (Total)	**SF-12**	Ware JE. 1996	57	G	12	2-6	Past 4 weeks	210				
Short Arthritis Assessment PROM (pain/physical activity/disability)	**SAS**	Wolfe F. 2004	60	G	4	11	Past week	2				
Medical Outcomes Study Short Form 36-Item (Role Physical)	**SF-36**	Ware JE. 1992	20	G	4	0-1	Past 4 weeks	989				
Shoulder Pain and Disability Index (Total: pain/disability)	**SPADI**	Roach KE. 1991	27	G	13	VAS/0-10	Past week	13				

Name (Domain)	Acronym / Name	First Author	REF	Generic/ Disease Specific	N of items	Response Options	Recall period	Use	Psychometric Summary Truth: Discrimination: Feasibility:			Overall
Western Ontario Osteoarthritis of the Shoulder Index (Total)	**WOOS**	Lo I. 2001	8	D	19	0-100	Past week	25				
Western Ontario Shoulder Instability Index (Total)	**WOSI**	Kirkley A. 1998	9	G	21	0-100	Past week	6				
World Health Organisation Disability Assessment Schedule –II (Total)	**WHODAS-II**	WHO 2004	30	G	36/32	5	Last 30 days	1				

Evidence: Strong; Moderate; Weak; Absent

Supplementary Table 12. General characteristics and Summary Psychometric Review of all versions of identified PROMs:

Other Psychological aspects, social and societal support. **(Personal/environmental factors)**

Name (Domain)	Acronym / Name	First Author	REF	Generic/ Disease Specific	N of items	Response Options	Recall period	Use	Psychometric Summary Truth:	Discrimination:	Feasibility:	Overall
Arthritis Impact Measurement Sc ales 2 (Social support)	**AIMS2**	Meenan R. 1992	1	D*	4	5	Past month	60				
Multidimensional Health Locus of Control (Personal beliefs)	**MHLC**	Wallston KA. 1978	61	G	18	6	Not specified	7				
Multidimensional Health Locus of Control (Internal HLC)	**MHLC**	Wallston KA. 1978	61	G	?	6	Not specified	7				
Multidimensional Health Locus of Control (Powerful others)	**MHLC**	Wallston KA. 1978	61	G	?	6	Not specified	7				
Multidimensional Health Locus of Control (Chance HLC)	**MHLC**	Wallston KA. 1978	61	G	?	6	Not specified	7				
Osteoarthritis of Knee Hip Quality of Life (social support)	**OAKHQOL**	Rat A-C. 2005	7	D	4	0-10	unknown	14				

Name (Domain)	Acronym / Name	First Author	REF	Generic/ Disease Specific	N of items	Response Options	Recall period	Use	Psychometric Summary Truth:	Discrimination:	Feasibility:	Overall
Patient Assessment of Chronic Illness Care (Total)	**PACIC**	Glasgow RE. 2005	62	G	20 (total)	5	Past 6 months	2				
Rehabilitation Patient Experiences Questionnaire (Total)	**Re-PEQ**	Grotle M. 2009	63	G	18	5	Previous rehab. care	1				
Rehabilitation Patient Experiences Questionnaire (rehab care and organization)	**Re-PEQ**	Grotle M. 2009	63	G	6	5	Previous rehab. care	1				
Rehabilitation Patient Experiences Questionnaire (information communication)	**Re-PEQ**	Grotle M. 2009	63	G	8	5	Previous rehab. care	1				
Rehabilitation Patient Experiences Questionnaire (availability of staff)	**Re-PEQ**	Grotle M. 2009	63	G	2	5	Previous rehab. care	1				
Rehabilitation Patient Experiences Questionnaire (social environment)	**Re-PEQ**	Grotle M. 2009	63	G	2	5	Previous rehab. care	1				

Name<br>(Domain)	Acronym<br>/ Name	First Author	REF	Generic/<br>Disease Specific	N<br>of items	Response<br>Options	Recall<br>period	Use	Psychometric Summary<br>Truth: Discrimination: Feasibility:	Overall
Medical Outcomes Study Short Form 36-Item<br>(Role Emotional)	**SF-36**	Ware JE.<br>1992	20	G	4	2	Past 4 weeks	989		

Evidence: Strong; Moderate; Weak; Absent

Supplementary Table 13. General characteristics and Summary Psychometric Review of all versions of identified PROMs:

Quality of life, including well being, and general health

Name (Domain)	Acronym / Name	First Author	REF	Generic/ Disease Specific	N of items	Response Options	Recall period	Use	Psychometric Summary<br>Truth:	Discrimination:	Feasibility:	Overall
Assessment of Quality of Life (psychological wellbeing)	**AQoL**	Hawthorne G. 1999	3	G	3	4	Last week	13				
Knee Disability and Osteoarthritis Outcome Score (Quality of life)	**KOOS**	Roos EM. 1998	15	D	4	5	Last week	886				
Medical Outcomes Study Short Form 36-Item (General Health)	**SF-36**	Ware JE. 1992	20	G			Past 4 weeks	989				
OA-Quality of Life Scale (HRQoL)	**OA-QoL**	Keenan AM. 2008	64	D	22	2	At the moment	1				
World Health Organization Quality of Life short version instrument (Total)	**WHOQOL-BREF**	WHO QoL Group 1998	65	G	26	5	Past 4 weeks	8				

Evidence: Strong; Moderate; Weak; Absent

Supplementary Table 14. General characteristics and Summary Psychometric Review of all versions of identified PROMs:

Health Utilities

Name (ICF)	Acronym / Name	First Author	REF	Generic/ Disease Specific	N of items	Response Options	Recall period	Use	Psychometric Summary Truth:	Discrimination:	Feasibility:	Overall
Assessment of Quality of Life (Utility)	AQoL	Hawthorne G. 1999	3	G	?	4	Last week	13				
EuroQol (Index)	EQ-5D -3L	Hurst N. 1994	66	G	5/1	3/100	Today	146				
Health Utilities Index-3 (Index)	HUI3	Feeny D 2002	67	G	8	Variable 5-6		10				
Medical Outcomes Study Short Form 6D (Total)	(SF-6D)	Brazier J. 2002	68	G	11	4-6	Past 4 weeks	25				
ICECAP-A	ICECAP_A	Al-Janabi, H 2012	70	G	5	4	At the moment	1				

Evidence: Strong; Moderate; Weak; Absent
