# Supplementary material for: Patient-reported outcome measures in osteoarthritis: a systematic search and review of their use and psychometric properties
Source: RMD Open. 2018 Dec 16;4(2):e000715. doi: 10.1136/rmdopen-2018-000715 (PMC6307597; doi:10.1136/rmdopen-2018-000715)
Supplement: Supplementary data [file rmdopen-2018-000715supp003.pdf]

Supplementary file 3

PROM specific references

1. Meenan RF, Mason JH, Anderson JJ, Guccione AA, Kazis LE. AIMS2. The content and properties of a revised and expanded Arthritis Impact Measurement Scales Health Status Questionnaire. Arthritis & Rheumatism. 1992;35(1):1-10

2. Guillemin F, Coste J, Pouchot J, Ghézail M, Bregeon C, Sany J. The AIMS2-SF. A short form of the arthritis impact measurement Scales 2. Arthritis & Rheumatism. 1997;40(7):1267-74.

3. Hawthorne G, Richardson J, Osborne R. The Assessment of Quality of Life (AQoL) instrument: a psychometric measure of health-related quality of life. Quality of Life Research. 1999;8(3):209-24.

4. Radloff LS. The CES-D Scale a self-report depression scale for research in the general population. Applied psychological measurement. 1977;1(3):385-401

5. Gaston JE, Vogl L. Psychometric properties of the general well-being index. Quality of Life Research. 2005;14(1):71-5.

6. Zigmond AS, Snaith RP. The hospital anxiety and depression scale. Acta psychiatr scand. 1983;67(6):361-70.

7. Rat A-C, Coste J, Pouchot J, Baumann M, Spitz E, Retel-Rude N, et al. OAKHQOL: a new instrument to measure quality of life in knee and hip osteoarthritis. Journal of clinical epidemiology. 2005;58(1):47-55.

8. Lo I, Griffin S, Kirkley A. The development of a disease-specific quality of life measurement tool for osteoarthritis of the shoulder: The Western Ontario Osteoarthritis of the Shoulder (WOOS) index. Osteoarthritis and Cartilage. 2001;9(8):771-8.

9. Kirkley A, Griffin S, McLintock H, Ng L. The Development and Evaluation of a Disease-Specific Quality of Life Measurement Tool for Shoulder Instability The Western Ontario Shoulder Instability Index (WOSI). The American Journal of Sports Medicine. 1998;26(6):764-72.

10. Meenan RF, Gertman PM, Mason JH. Measuring health status in arthritis. Arthritis & Rheumatism. 1980;23(2):146-52.

11. Bellamy N, Campbell J, Haraoui B, Buchbinder R, Hobby K, Roth J, et al. Dimensionality and clinical importance of pain and disability in hand osteoarthritis: Development of the Australian/Canadian (AUSCAN) Osteoarthritis Hand Index. Osteoarthritis and Cartilage. 2002;10(11):855-62

12. Cleeland C, Ryan K. Pain assessment: global use of the Brief Pain Inventory. Annals of the Academy of Medicine, Singapore. 1994;23(2):129-38.

13. Budiman-Mak E, Conrad KJ, Roach KE. The Foot Function Index: a measure of foot pain and disability. Journal of clinical epidemiology. 1991;44(6):561-70.

14. Klässbo M, Larsson E, Mannevik E. Hip disability and osteoarthritis outcome score An extension of the Western Ontario and McMaster Universities Osteoarthritis Index. Scandinavian journal of rheumatology. 2003;32(1):46-51.

15. Roos EM, Roos HP, Lohmander LS, Ekdahl C, Beynnon BD. Knee Injury and Osteoarthritis Outcome Score (KOOS)—development of a self-administered outcome measure. Journal of Orthopaedic & Sports Physical Therapy. 1998;28(2):88-96.

16. Lequesne M. The algofunctional indices for hip and knee osteoarthritis. The Journal of rheumatology. 1997;24(4):779-81.

17. Lequesne M, Mery C, Samson M, Gerard P. Indexes of severity for osteoarthritis of the hip and knee: validation-value in comparison with other assessment tests. Scandinavian journal of rheumatology. 1987;16(S65):85-9.

18. Melzack R. The McGill Pain Questionnaire: major properties and scoring methods. Pain. 1975;1(3):277-99.

19. Hawker G, Davis A, French M, Cibere J, Jordan J, March L, et al. Development and preliminary psychometric testing of a new OA pain measure–an OARSI/OMERACT initiative. Osteoarthritis and Cartilage. 2008;16(4):409-14.

20. Ware Jr JE, Sherbourne CD. The MOS 36-item short-form health survey (SF-36): I. Conceptual framework and item selection. Medical care. 1992:473-83.

21. Hunt, SM; McEwen, J; McKenna, SP. Measuring health status: A new tool for clinicians and epidemiologists. The Journal of the Royal College of General Practitioners 1985;35(273):185–8.

22. Dawson J, Doll H, Boller I, Fitzpatrick R, Little C, Rees J, et al. The development and validation of a patient-reported questionnaire to assess outcomes of elbow surgery. Journal of Bone & Joint Surgery, British Volume. 2008;90(4):466-73.

23. Sullivan MJ, Bishop SR, Pivik J. The pain catastrophizing scale: development and validation. Psychological assessment. 1995;7(4):5.

24. MacDermid JC. Outcome evaluation in patients with elbow pathology: issues in instrument development and evaluation. Journal of Hand Therapy. 2001;14(2):105-14.

25. MacDermid JC. Development of a PROM for patient rating of wrist pain and disability. Journal of Hand Therapy. 1996;9(2):178.

26. Leeb B, Sautner J, Andel I, Rintelen B. SACRAH: a score for assessment and quantification of chronic rheumatic affections of the hands. Rheumatology. 2003;42(10):1173-8.

27. Roach KE, Budiman-Mak E, Songsiridej N, Lertratanakul Y. Development of a shoulder pain and disability index. Arthritis & Rheumatism. 1991;4(4):143-9.

28. Bellamy N. Validation study of WOMAC: a health status instrument for measuring clinically-important patient-relevant outcomes following total hip or knee arthroplasty in osteoarthritis. J Orthop Rheumatol. 1988;1:95-108.

29. Kosinski M, Janagap CC, Gajria K, Schein J. Psychometric testing and validation of the Chronic Pain Sleep Inventory. Clinical therapeutics. 2007;29(11):2562-77.

30. World Health Organization (WHO). WHODAS II - Disability Assessment Schedule Training Manual: A guide to administration [Internet]. Geneve: WHO; 2004.

31. Baecke J, Burema J, Frijters J. A short questionnaire for the measurement of habitual physical activity in epidemiological studies. The American journal of clinical nutrition. 1982;36(5):936-42.

32. Duruöz M, Poiraudeau S, Fermanian J, Menkes C, Amor B, Dougados M, et al. Development and validation of a rheumatoid hand functional disability PROM that assesses functional handicap. The Journal of rheumatology. 1996;23(7):1167-72.

33. Saleh KJ, Mulhall KJ, Bershadsky B, Ghomrawi HM, White LE, Buyea CM, et al. Development and validation of a lower-extremity activity PROM. The Journal of Bone & Joint Surgery. 2005;87(9):1985-94.

34 Gignac MA, Sutton D, Badley EM. Arthritis symptoms, the work environment, and the future: measuring perceived job strain among employed persons with arthritis. Arthritis care & research. 2007;57(5):738-47.

35. Gilworth G, Chamberlain MA, Harvey A, Woodhouse A, Smith J, Smyth MG, et al. Development of a work instability PROM for rheumatoid arthritis. Arthritis care & research. 2003;49(3):349-54.

36. Lerner D, Amick III BC, Rogers WH, Malspeis S, Bungay K, Cynn D. The work limitations questionnaire. Medical care. 2001;39(1):72-85.

37. Gignac MA. Arthritis and employment: an examination of behavioral coping efforts to manage workplace activity limitations. Arthritis care & research. 2005;53(3):328-36.

38. Jette AM, Haley SM, Coster WJ, Kooyoomjian JT, Levenson S, Heeren T, et al. Late life function and disability instrument I. Development and evaluation of the disability component. The Journals of Gerontology Series A: Biological Sciences and Medical Sciences. 2002;57(4):M209-M16.

39. Hudak PL, Amadio PC, Bombardier C. Development of an upper extremity outcome measure: the DASH. Am J Ind Med. 1996;29(6):602-8.

40. Fries JF, Spitz P, Kraines RG, Holman HR. Measurement of patient outcome in arthritis. Arthritis & Rheumatism. 1980;23(2):137-45.

41. Davis A, Perruccio A, Canizares M, Tennant A, Hawker G, Conaghan P, et al. The development of a short measure of physical function for hip OA HOOS-Physical Function Shortform (HOOS-PS): an OARSI/OMERACT initiative. Osteoarthritis and Cartilage. 2008;16(5):551-9.

42. Fix AJ, Daughton D. Human activity profile: Professional manual: Psychological Assessment Resources; 1988.

43. Akinpelu A, Odole A, Adegoke B, Adeyini A. Development and initial validation of the ibadan knee/hip osteoarthritis outcome measure. South African Journal of Physiotherapy. 2007;63(2):3-8.

44. Perruccio AV, Lohmander LS, Canizares M, Tennant A, Hawker GA, Conaghan PG, et al. The development of a short measure of physical function for knee OA KOOS-Physical Function Shortform (KOOS-PS)–an OARSI/OMERACT initiative. Osteoarthritis and Cartilage. 2008;16(5):542-50.

45. Irrgang JJ, Snyder-Mackler L, Wainner RS, Fu FH, HARNER CD. Development of a Patient-Reported Measure of Function of the Knee*. The Journal of Bone & Joint Surgery. 1998;80(8):1132-45.

46. Haley SM, Jette AM, Coster WJ, Kooyoomjian JT, Levenson S, Heeren T, et al. Late Life Function and Disability Instrument II. Development and Evaluation of the Function Component. The Journals of Gerontology Series A: Biological Sciences and Medical Sciences. 2002;57(4):M217-M22

47. Binkley JM, Stratford PW, Lott SA, Riddle DL. The Lower Extremity Functional PROM (LEFS): PROM development, measurement properties, and clinical application. Physical therapy. 1999;79(4):371-83.

48. Tugwell P, Bombardier C, Buchanan W, Goldsmith C, Grace E, Hanna B. The MACTAR Patient Preference Disability Questionnaire--an individualized functional priority approach for assessing improvement in physical disability in clinical trials in rheumatoid arthritis. The Journal of rheumatology. 1987;14(3):446-51.

49. Chung KC, Pillsbury MS, Walters MR, Hayward RA. Reliability and validity testing of the Michigan Hand Outcomes Questionnaire. The Journal of hand surgery. 1998;23(4):575-87.

50. Pincus T, Summey JA, Soraci SA, Wallston KA, Hummon NP. Assessment of patient satisfaction in activities of daily living using a modified stanford health assessment questionnaire. Arthritis and Rheumatism 1983; 26: 1346-53.

51. Pincus T, Yazici Y, Bergman M. Development of a multi-dimensional health assessment questionnaire (MDHAQ) for the infrastructure of standard clinical care. Clinical and experimental rheumatology. 2005;23(5):S19 .

52. Fairbank JC, Pynsent PB. The Oswestry Disability Index. Spine 2000 Nov 15;25(22):2940-52.

53. Dawson J, Fitzpatrick R, Carr A, Murray D. Questionnaire on the perceptions of patients about total hip replacement. Journal of Bone & Joint Surgery, British Volume. 1996;78(2):185-90.

54. Dawson J, Fitzpatrick R, Murray D, Carr A. Questionnaire on the perceptions of patients about total knee replacement. Journal of Bone & Joint Surgery, British Volume. 1998;80(1):63-9

55. Dawson J, Fitzpatrick R, Carr A. Questionnaire on the perceptions of patients about shoulder surgery. Journal of Bone & Joint Surgery, British Volume. 1996;78(4):593-600.

56. Beaton DE, Wright JG, Katz JN. Upper Extremity Collaborative Group. Development of the QuickDASH: comparison of three item-reduction approaches. The Journal of Bone and Joint Surgery American Volume 2005;87:1038-46.

57. Ware Jr JE, Kosinski M, Keller SD. A 12-Item Short-Form Health Survey: construction of PROMs and preliminary tests of reliability and validity. Medical care. 1996;34(3):220-33.

58. Devins GM, Binik YM, Hutchinson TA, Hollomby DJ, Barré PE, Guttmann RD. The emotional impact of end-stage renal disease: Importance of patients' perceptions of intrusiveness and control. The International Journal of Psychiatry in Medicine. 1984;13(4):327-43.

59. Pallant JF, Misajon R, Bennett E, Manderson L. Measuring the impact and distress of health problems from the individual's perspective: development of the Perceived Impact of Problem Profile (PIPP). Health and quality of life outcomes. 2006;4(1):36.

60. Wolfe F, Michaud K, Kahler K, Omar M. The Short Arthritis Assessment PROM: a brief assessment questionnaire for rapid evaluation of arthritis severity in research and clinical practice. The Journal of rheumatology. 2004;31(12):2472-9.

61. Wallston KA, Wallston BS, DeVellis R. Development of the multidimensional health locus of control (MHLC) PROMs. Health Education & Behavior. 1978;6(1):160-70.

62. Glasgow RE, Wagner EH, Schaefer J, Mahoney LD, Reid RJ, Greene SM. Development and validation of the patient assessment of chronic illness care (PACIC). Medical care. 2005;43(5):436-44.

63. Grotle M, Garratt A, Løchting I, Kjeken I, Klokkerud M, Uhlig T, et al. Development of the rehabilitation patient experiences questionnaire: data quality, reliability and validity in patients with rheumatic diseases. Journal of rehabilitation medicine. 2009;41(7):576-81.

64. Keenan AM, Mckenna SP, Doward LC, Conaghan PG, Emery P, Tennant A. Development and validation of a needs-based quality of life instrument for osteoarthritis. Arthritis care & research. 2008;59(6):841-8.

65.WHOQoL Group. Development of the World Health Organization WHOQOL-BREF quality of life assessment. Psychological medicine. 1998;28(03):551-8.

66. Hurst N, Jobanputra P, Hunter M, Lambert M, Lochhead A, Brown H. Validity of euroqol—a generic health status instrument—in patients with rheumatoid arthritis economic and health outcomes research group. Rheumatology. 1994;33(7):655-62.

67. Feeny D, Furlong W, Torrance GW, Goldsmith CH, Zhu Z, DePauw S, Denton M, Boyle M. Multi-attribute and single-attribute utility functions for the Health Utilities Index Mark 3 system. Med Care. 2002;40(2):113–28.

68. Brazier J, Roberts J, Deverill M. The estimation of a preference-based measure of health from the SF-36. Journal of health economics. 2002;21(2):271-92.

69. Gignac MA, Sutton D, Badley EM. Reexamining the arthritis-employment interface: perceptions of arthritis-work spillover among employed adults. Arthritis Rheum. 2006;55(2):233-40.

70 Al-Janabi, H., Flynn, T. N., & Coast, J. Development of a self-report measure of capability wellbeing for adults: The ICECAP-A. Quality of Life Research. 2012;21(1):167–176.

71 Singh JA, Schleck C, Harmsen WS, Lewallen DG. Validation of the Mayo Hip Score: construct validity, reliability and responsiveness to change. BMC Musculoskelet Disord. 2016;17(39):1-7.

72. Dreiser RL, Maheu E, Guillou GB, Caspard H, Grouin JM. Validation of an algofunctional index for osteoarthritis of the hand. Rev Rhum (Engl ed.). 1995; 62(Suppl 1):43S-53S.

73. Reilly MC, Zbrozek AS, Dukes EM. The validity and reproducibility of a work productivity and activity instrument. Pharmacoeconomics. 1993;4:353–65.

74 Amtmann DA, Cook KF, Jensen MP, Chen W-H, Choi SW, Revicki D, Cella D, Rothrock N, Keefe F, Callahan L, Lai J-S. Development of a PROMIS item bank to measure pain interference. Pain. 2010;150(1):173-82.

75. Choi SW, Reise SP, Pilkonis PA, Hays RD, Cella D. Efficiency of static a computer adaptive short forms compared to full-length measures of depressive symptoms. Qual Life Res. 2010;19(1):125-36.

76. Hays RD, Spritzer KL, Fries JF, Krishnan E: Responsiveness and minimally important difference for the Patient Reported Outcomes Measurement Information System (PROMIS) 20-item physical functioning short form in a prospective observational study of rheumatoid arthritis. Ann Rheum Dis. 2013;74:1-4.

77. Pilkonis PA, Choi SW, Reise SP, Stover AM, Riley WT, Cella D, et al. Item Banks for Measuring Emotional Distress From the Patient-Reported Outcomes Measurement Information System (PROMIS®): Depression, Anxiety, and Anger. Assessment. 2011;18:263–83.

78. Nordenskiöld U, Grimby G, Hedberg M, Wright B, Linacre JM. The structure of an instrument for assessing the effects of assistive devices and altered working methods in women with rheumatoid arthritis. Arthritis Care Res. 1996;9(5):358-67.
